# Internalizing and Externalizing Symptoms in Adolescents With and Without Experiences of Physical Parental Violence, a Latent Profile Analysis on Violence Resilience

**DOI:** 10.3389/fpsyg.2022.824543

**Published:** 2022-03-31

**Authors:** Dilan Aksoy, Céline A. Favre, Clarissa Janousch, Beyhan Ertanir

**Affiliations:** Department of Research and Development, School of Education, University of Applied Sciences and Arts Northwestern Switzerland, Windisch, Switzerland

**Keywords:** latent profiles, internalizing, externalizing, physical family violence, maltreated youth, violence resilience, psychopathology

## Abstract

Questionnaire data from a cross-sectional study on social resilience in adolescence, with a sample of *N* = 1,974 Swiss seventh grade high school students ages 12–14 (*M* = 11.76; *SD* = 0.65) was used to identify and compare violence resilience profiles. Person-centered latent profile analysis (LPA) was applied and allowed for the grouping of adolescents into profiles of internalizing (depression/anxiety, dissociation) and externalizing symptoms (peer aggression, peer victimization, classroom disruption) and differentiation of adolescents with (*n* = 403) and without (*n* = 1,571) physical parental violence experiences. Subsequently, a multinomial logistic regression analysis was conducted to further investigate the sociodemographic predictors of violence resilience profiles. With LPA, we identified four distinct profiles for both adolescent groups (with and without parental physical violence experiences). The results showed three particularly burdened profiles of adolescents, one with *higher externalizing* and one with *higher internalizing* symptoms, which did not occur simultaneously to the same extent. Furthermore, the third profile contained adolescents with both elevated internalizing and externalizing symptoms, the *comorbid* profile. The fourth profile consisted of the majority of adolescents, who exhibited little or no internalizing and externalizing symptoms, the so-called *no/low symptomatic* profile. A differentiated view of the symptoms can create added value regarding the understanding of violence resilience. Moreover, in the multinomial logistic regression, significant associations were found between the profiles and adolescents’ gender in the group of adolescents with parental physical violence experiences, but none were found in relation to sociocultural status and migration background.

## Introduction

Youths are exposed to numerous risk factors. A major risk factor for adolescents is maltreatment, which can be associated with short-term (Kapella, 2011; Straus et al., 2017; Enzmann et al., 2018; Kassis et al., 2018) and long-term consequences (Chapman et al., 2004; Norman et al., 2012; Li et al., 2016; Maneta et al., 2017). Adolescents’ exposure to family violence as a form of maltreatment, such as experiencing physical violence by parents, is a common burden for adolescents (Straus et al., 2017). Studies show a prevalence of 20–25% for the European Union (Kapella, 2011; Enzmann et al., 2018; Kassis et al., 2018) and North America (Moody et al., 2018) and 19% for Switzerland (Enzmann et al., 2018).

Experiencing family violence can lead to increased levels of psychopathological symptoms (Cicchetti and Toth, 2015) in adolescents, such as which can be categorized as externalizing and internalizing symptoms. Findings suggest that externalizing symptoms in individuals with parental violence experiences predominantly manifest at a younger age and decrease over time. In contrast, researchers have reported that internalizing symptoms increase during childhood and are particularly prevalent in adolescence (Riina et al., 2014; Wiggins et al., 2015). Therefore, adolescence is an especially important developmental stage regarding the consequences of parental violence experiences.

For example, disruptive behavior (Cicchetti and Toth, 2015) and aggressive behavior (Evans et al., 2008; Baydar and Akcinar, 2018; Davis et al., 2020) are described as externalizing symptoms. Maltreated children are understood to judge more hastily the attribution of hostile intentions, show more aggressive reactions, and consider aggression an appropriate response (Teisl and Cicchetti, 2008). As some studies have shown, these mechanisms can lead to a higher risk of re-victimization after parental physical violence (Shields and Cicchetti, 2001; Yodprang et al., 2009), especially during the middle school years (Hymel and Swearer, 2015). Moreover, Benedini et al. (2016) demonstrated that people who were physically abused as children have a higher risk of being victimized by peers as adolescents, a process that they termed “the cycle of victimization.”

Further studies showed that maltreated youths show internalizing symptoms, such as high levels of depression (see Asgeirsdottir et al., 2010; Kassis et al., 2013a; Runyon et al., 2014; Tlapek et al., 2017), anxiety (Rehan et al., 2017; Gerin et al., 2019; Guo et al., 2021), and dissociation (Macfie et al., 2001; Mariscal, 2020; Tschoeke et al., 2021). Cicchetti and Toth (2015) noted that physical abuse, in particular, has proven to be predictive of the development of dissociation at the clinical level, as possibly the severest deficit in integrating oneself.

One question that researchers from various disciplines are addressing is how certain adolescents can adapt successfully despite experiencing parental violence (Afifi and MacMillan, 2011; Yule et al., 2019; Yoon et al., 2019). Important to this question is positive adaptation *despite* family violence, which has become a basic tenet of resilience research and is evident in Masten’s (2014) definition of resilience. The “capacity of a system to adapt successfully to disturbances that threaten the viability, function, or development of the system” is currently widely referred to as resilience (Masten, 2014, p. 6). Resilience researchers, then, try to identify how risk factors can be reduced and how positive development can be promoted (Masten, 2019). Various understandings and conceptualizations of resilience have evolved depending on the risk factors youth face and the strength of the link to psychological health. We cannot say whether we can describe someone as resilient in general terms; this description depends on the particular risk factors to which an individual is exposed. Therefore, it is important to look at risk-specific resilience in its context. When youths are facing severe risk factors, rather than defining resilience as excellent adjustment, it is appropriate to define the criterion of resilient development as the absence of severe psychopathology (Luthar et al., 2015).

In the current violence resilience literature, a lack of research is apparent in operationalizing violence resilience in a non-dichotomous way. Person-centered analysis, such as latent profile analysis (LPA), has gained increasing attention in empirical research to locate homogeneous subgroups within heterogenous samples and is therefore increasingly used in analysis of youths with histories of childhood adversity (Rebbe et al., 2017; Lanier et al., 2018; Mariscal, 2020). A few person-centered studies that have examined psychopathology symptoms have been able to indicate that adolescents cannot be clearly categorized into adolescents with externalizing or internalizing symptoms, but that comorbidities exist (Gallitto et al., 2017; Duprey et al., 2020). Duprey et al. (2020) moreover embedded their profiles in the resilience framework. However, there are certain gaps in the existing studies that need to be filled. Some have used only internalizing symptoms (Gallitto et al., 2017), have not measured a history of violence (Berona et al., 2017), or have used only two indicators as internalizing or externalizing symptoms (Duprey et al., 2020). Furthermore, the studies had criterion-based samples that were oversampled with subjects who presented symptoms.

The aim of this paper is therefore to identify person-centered violence resilience profiles, with a focus on externalizing and internalizing symptoms that differentiate resilience profiles and do not conceptualize resilience outcomes as a dichotomy (i.e., either resilient or non-resilient). In addition, the neglected individual characteristics such as gender, SES and migration background are taken into account, which allows us to approach the detection of social inequalities (Bottrell, 2009; Yule et al., 2019) as well as the comparison to a group without physical parental violence (Yule et al., 2019).

## Violence Resilience as a Risk-Specific Theoretical Framework

Several authors who have been working on the topic of resilience are considered important founders of the theory and conceptualize resilience as a process that can change over time (Garmezy, 1991; Rutter, 1993; Luthar et al., 2000; Masten, 2001; Ungar, 2004). Whether someone is resilient in the face of adversity can consequently change with shifting circumstances regarding stressors, the social environment, and the individual, with new strengths and vulnerabilities emerging (Luthar and Brown, 2007; Ungar et al., 2013). In empirical resilience research, it is noticeable that resilience goes hand in hand with adversity and positive adaptation. How exactly adversity and positive adaption are operationalized, however, differs significantly. Systematic reviews and meta-analyses of resilience literature are difficult to obtain because the concept of resilience is defined and operationalized in very different ways (Masten and Barnes, 2018). Resilience theory can therefore be helpful in identifying and potentially supporting at-risk individuals but must be specifically focused on the risk factors and time periods (Vanderbilt-Adriance and Shaw, 2008) to derive target-oriented results and helpful measures.

A large body of evidence already demonstrates that parental violence has a devastating impact on adolescents’ development. Inconsistency has emerged, however, regarding which indicators we should use to measure violence resilience. Meng et al. (2018) discovered this inconsistency, which resulted in their inability to conduct a meta-analysis because the data were too different. The authors concluded that the studies varied in their study characteristics, such as their conceptualization of resilience, measurement outcomes, and/or sample characteristics (e.g., sample size, form of maltreatment) (Meng et al., 2018). Violence resilience focuses on individuals that demonstrate resilience following maltreatment experiences and can be conceptualized in various ways. Yoon et al. (2019) showed in their review that authors either used resilience-specific measures and scales that measured how well one is equipped to “bounce back” after adversity, a multidomain composite resilience score of functioning domains (e.g., academic, social, behavioral), or outcomes related to adaptive functioning and is often conceptualized as “reaching normative developmental milestones after maltreatment” (Yoon et al., 2019, p. 544). The first perspective conceptualizes violence resilience as a trait, which many resilience researchers advise against (Rutter, 2013; Wright et al., 2013), as it can lead to victim-blaming. Wright et al. (2013) noted in *The Handbook of Resilience in Children* that a focus on resilience as a trait blames children for not being able to adapt successfully and underestimates the comprehensive role of context in individual resilience. Overwhelming social stressors, chronic adversity faced by many children, as well as family, school, neighborhood, community, and cultural embeddedness that influence children’s resilience are not considered. The second perspective presents resilience as a socioecological resource (internal and external resources that improve individual well-being) as protective factors, often taken together in the form of a composite score of various positive and negative indicators. The third perspective focuses predominantly on the absence of psychopathology, as review studies showed that two thirds of the studies involved measured psychopathology as an indicator for adaptive functioning (Yule et al., 2019). In this regard, Luthar et al. (2015) notes that the indicators used to operationalize positive/successful adjustment must be both developmentally appropriate and relevant to the risk condition under study. She notes that decisions about the stringency of the criteria for what constitutes positive adjustment must also be informed by the severity of the risks being studied. When researching adolescents facing severe risk factors, it is appropriate to define the criterion of resilient development as the absence of severe psychopathology, rather than defining it as excellent adjustment (Luthar et al., 2015). Parental violence is seen as a major form of childhood adversity (Kapella, 2011; Straus et al., 2017; Enzmann et al., 2018; Kassis et al., 2018); therefore, this paper focuses on the absence of psychopathology as a determinant of violence resilience profiles, following existing studies (Collishaw et al., 2007; Dang, 2014; Go et al., 2017; Kassis et al., 2018; Duprey et al., 2020). Based on the research findings derived above, violence resilience in this paper refers to resilience to externalizing and internalizing symptoms.

Furthermore, the question arises of whether a dichotomous operationalization of resilience profiles (i.e., resilient vs. non-resilient) differentiates individuals sufficiently. Although resilience theory is multisystemic and process-oriented, the operationalization of resilience, at least in the parental-violence domain, turns out to be mainly dichotomous. Particularly regarding the processual nature of violence resilience, nuances in changes over time are lost in a dichotomous operationalization of resilience outcomes. This operationalization also requires researchers to establish clear criteria for resilience, as most researchers are left with the task of defining average or above average functioning (Walsh et al., 2010). Most studies that assess violence resilience outcomes often focus on dichotomous outcomes (e.g., DuMont et al., 2007; Nishimi et al., 2020) or on composite scores (e.g., Cicchetti and Rogosch, 2012; Sexton et al., 2015; Arslan, 2016). For example, DuMont et al. (2007) considered youths resilient if they were successful in four out of five domains, ranging from graduating from high school to lack of psychiatric diagnoses. Although the researchers considered multiple domains, the variable for resilience itself was dichotomized and therefore divided the youths into resilient and non-resilient categories. Different criteria, in turn, lead to different outcomes, more complicating the comparison of these outcomes, and do not provide space for youths who cannot or should not be clearly labeled as resilient or non-resilient. As one of the less common examples, Kassis et al. (2013b,2015) operationalized violence resilience non-dichotomously and showed that youths can find their place between the extremes of resilient and non-resilient and that the absolute achievement of resilience is not necessarily the only positive form of development, but a continuum-based resilience can be a useful alternative to the dichotomous operationalization. In a cross-sectional study of family violence and resilience in a random sample of 5,149 middle school students in Europe, the researchers found that 31% of youths were resilient, 28.3% near-resilient (mid-level scores for violence perpetration and/or depression symptoms), and 40.6% non-resilient (Kassis et al., 2013b). In contrast, Walsh et al. (2010) showed that although 40% of 1,041 adolescents (11–15 years of age) with experiences of maltreatment were resilient in at least one domain (either externalizing symptoms, internalizing symptoms, or educational success), only 16% were assessed as competent in all three domains, and 24% of the adolescents were not resilient in any of the three domains.

### Person-Centered Approaches in Violence Resilience Research

One way to break down dichotomies in classification is through person-centered methods. Variable-centered methods focus on associations between variables across individuals whereas person-centered approaches focus on profiles within individuals (Rivera et al., 2018). Person-centered analysis, such as LPA, has gained increasing attention in empirical research and is therefore increasingly used in analysis of youths with histories of childhood adversity (Rebbe et al., 2017; Lanier et al., 2018; Mariscal, 2020). In existing studies, researchers have mostly focused on polyvictimization or multiple risk categories, such as various forms of adversity (Parra et al., 2006; Bowen et al., 2007; Hazen et al., 2009; Rivera et al., 2018; Nelon et al., 2019) or exposure to violence occurring between parents (McDonald et al., 2016a,b). A few studies dealing with psychopathological symptom profiles are now briefly presented.

Gallitto et al. (2017) conducted an LPA and identified three trauma symptom profiles in 479 child-welfare-involved adolescents (ages 13–17) with child maltreatment experiences, where more than half of the adolescents (59%) were in the minimal symptom group and the others fell into the moderate (30%) and severe (11%) symptom groups. Researchers conceptualized maltreatment in the study using various experiences of violence, including physical violence. As indicators of internalizing symptoms they measured anxiety, depression, anger, posttraumatic stress, dissociation, and sexual problems. They used no externalizing symptoms as indicators. In a study by Berona et al. (2017), they analyzed 433 acutely suicidal, psychiatrically hospitalized adolescents’ psychopathology profiles through LPA and identified four profiles. Of the participants, 43% fit the subclinical profile, 29% fit the internalizing profile, 17% fit the moderately dysregulated profile, and 11% fit the severely dysregulated profile. In the two dysregulated profiles, the Anxious/Depressed, Attention Problems, and Aggressive Behavior indicators had elevated mean levels on a clinical level. In the internalizing profile only the Anxious/Depressed indicators had elevated means levels on a clinical level. And in the subclinical profile no indicator had elevated means on a clinical level. The researchers did not assess a history of maltreatment. In another study, Duprey et al. (2020) focused on longitudinal profiles following risk factors, with a sample of *N* = 1,314 children and adolescents aged 4–14 and identified using the Bivariate Growth Curve Model’s four profiles. The profiles were named the high comorbidity class (6%), the high externalizing class (8%), the moderating and decreasing class (3%), and the low symptomology class (82%), the latter labeled resilient pathway. They found that severe physical abuse increased the adolescents’ risk of inclusion in the high externalizing or high comorbidity class rather than the moderate or decreasing class. The researchers confirmed the findings of Willner et al. (2016), who also found a comorbid group that was particularly at risk for exhibiting suicidal behaviors. Willner et al. (2016), in their person-centered longitudinal study on children through second grade across five measurement time points with a sample of *N* = 336, found comorbid (48%), internalizing (19–23%), externalizing (21–22%), and well-adjusted (7–11%) groups. The few studies that have addressed the issue of psychopathology symptoms resulting from forms of maltreatment or more general adversity seem to locate similar profiles with different expressions of internalizing and externalizing symptoms.

### Gender, Migration Background and Socioeconomic Status as Predictors of Violence Resilience Profiles

As Yoon et al. (2019) highlighted in their review, which focused on resilience in the context of child maltreatment, individuals’ characteristics, such as race/ethnicity, socioeconomic status, gender identity, and sexual orientation, have received only limited attention. Therefore, only in very few violence resilience studies have researchers examined the role of gender, socioeconomic status (SES), or migration background. Defining a person with a migration background is a complex issue, as the context of the country and its migration policies play an important role in the meaning of the term. In the Swiss context, people have a migration background if they are foreign nationals or naturalized citizens. Except for those born in Switzerland and whose parents were both born in Switzerland, as well as individuals with citizenship at birth whose parents were both born abroad (Federal Statistical Office, 2020).

Studies examining individual characteristics related to resilience show mixed results. For example, Flores et al. (2005) and Collishaw et al. (2007) found no gender-based differences between resilient and non-resilient individuals whereas Flores et al. (2005) found an association between low SES and non-resilience in Latino youth with violence experiences. In contrast, Kassis et al., 2013b showed that being female and having a high SES increased youths’ odds of being in the resilient youth group, but they cautioned that we must consider the low associations. Migration background made no difference in the resilience level. In comparison, Duprey et al. (2020) showed that adolescents who identified as African-American would more likely be in the high externalizing group or the low symptomology group than in the comorbidity group. SES and gender did not have a direct effect on group membership.

In the context of internalizing and externalizing symptoms, Belhadj Kouider et al. (2014) found in their systematic review of emotional and behavioral problems in migrant children and adolescents in Europe, independent of experiences of violence, that childhood with a migration background in Europe was often considered a risk factor for internalizing problem behavior. Migration status itself was often postulated as a risk factor for children’s mental health (especially first-generation migrants). The prevalence rate for externalizing problem behavior was comparable between Native and migrant children. Furthermore, the study showed that internalizing problem behavior occurred more often in girls than in boys, independent of migration background.

## Overview of the Present Study

To investigate conceptual and methodological gaps in research on violence resilience, in the present study, we used a combination of person- and variable-centered analysis, as Masten and Barnes (2018) consider such a combination particularly valuable in resilience research. We intended to take a person-centered approach to violence resilience to involve the heretofore neglected aspects of individual differences and to compare youths with and without parental physical violence (Yule et al., 2019). In the context of research findings on resilience despite parental experiences of violence, studies have often focused on what protective factors lead to the absence of psychopathological symptoms (see Yule et al., 2019). However, the present study takes a step back and examines the occurrence of psychopathological symptom profiles in adolescents with and without experiences of parental violence. This is consistent with theoretical considerations by Luthar et al. (2015), as the criterion that defines resilience should always be chosen in the context of the risk factor(s). The aim is to find out how the typical internalizing and externalizing symptoms display themselves in adolescents who have experienced violence. Building on the existing body of research on psychopathological symptoms following experiences of violence, the following explorative research questions and hypotheses can be formulated:

1) How many and what kind of non-dichotomous profiles of externalizing and internalizing symptoms can we identify using LPA, and how are they composed?

Based on the findings of Willner et al. (2016), Berona et al. (2017), and Duprey et al. (2020) on person-centered psychopathology outcomes with internalizing and externalizing symptoms, there is an indication that adolescents with experiences of parental violence belong to four profiles. Although the three studies have differences in design and sample, there are similarities in terms of the profiles or groups found. Berona et al. (2017) only considered internalizing symptomatology and came up with three profiles or groups. Given the different designs and samples of the studies, and the resulting differences in content of the profiles, there are only two profiles that appear across all studies, one with low-level symptoms and one comorbid profile. Therefore, considering both internalizing and externalizing indicators, our first hypothesis was to find four profiles in the present study as well, with at least one low-level symptom profile and one comorbid profile. We expected that the other two profiles would consist of increased internalizing, increased externalizing, or further comorbid profiles.

2) Do differences exist in externalizing and internalizing symptom profiles between adolescents with and without experiences of parental physical violence?

Based on the high susceptibility of youths with parental violence experiences to psychopathology (see e.g., Cicchetti and Toth, 2015), we expected that differences exist in symptom profiles for experiences of parental physical violence in comparison to the profiles without experiences of parental physical violence. We therefore hypothesized that youths without experiences of parental physical violence would have lower symptom profiles than those with.

3) Do gender, migration background, and sociocultural status predict the profile membership?

The findings of Kassis et al. (2013b), Benedini et al. (2016), Gallitto et al. (2017), and Duprey et al. (2020) show different results concerning psychopathology or resilience regarding gender and migration background, which may indicate minor differences between them. Flores et al. (2005) and Kassis et al., 2013b found significant associations between SES and resilience, indicating that as SES increases, the odds of being resilient increase. Flores et al. (2005) and Duprey et al. (2020) found significant relationships between migration background and non-resiliency. Therefore, we hypothesized that with low SES and migration background, the chances of being violence resilient decrease.

## Materials and Methods

### Participants

The analyzed data comes from a cross-sectional sample of a broader study on adolescents’ resilience conducted in the autumn of 2020. The random sample consisted of 1,974 seventh grade middle school students from Switzerland as well as 1,000 (51.2%) assigned females and 952 (48.8%) assigned males who anonymously completed an online questionnaire. We obtained consent forms from students and their caregivers and provided no incentives. The research ethics committee at the School of Education, FHNW in Switzerland, authorized the project. On the day of the study, the research team members gave a short oral introduction about the study to the students who were present in the 141 participating classes. Participating students completed the questionnaire in about 60 min. The sample’s overall average age was *M* = 11.76 (*SD* = 0.65). Of the participating students, 1,029 (52.6%) were Swiss citizens.

### Externalizing and Internalizing Latent Profile Indicators

#### Physical Parental Violence

We assessed *Physical parental violence* using five items that were part of the Alabama Parenting Questionnaire (APQ; Frick, 1991). The five items focus on the subdimensions corporal punishment and physical aggression, with a focus on serious physical parental abuse. The items were rated on a 5-point Likert scale ranging from 1 = *never* to 5 = *always*, where higher scores indicate a greater frequency of physical parental abuse. Cronbach’s alpha [α] was 0.83, indicating good internal consistency. Items included “My parents beat me up when I have done something wrong” and “My parents beat me up so severely that I had to go and see a doctor or rush to the hospital.” Participants with physical abuse scores higher than 1 (1 = *never*) were categorized as 1 (*serious physical parental violence)* and adolescents with a mean score of 1 over all five items were categorized as 0 (*no serious physical parental violence*).

#### Symptoms of Depression

We assessed *Symptoms of anxiety/depression* using 24 items that were part of the Hopkins Symptom Checklist (Derogatis et al., 1974). From the original 25-item scale, we left out one item, *Loss of sexual interest or pleasure*, because of the participants’ young age. We rated the items on a 4-point Likert scale ranging from 1 = *not at all* to 4 = *extremely*, where higher scores indicated a greater severity of anxiety and depression symptoms (α = 0.96). Items included “I feel fear” and “Thoughts of ending my life.” For the LPA, we calculated the mean score.

#### Symptoms of Dissociation

We measured the items for assessing dissociation (Colizzi et al., 2015) as a disruption or discontinuity of consciousness using a four-item short scale (DSS-4) of the *Dissociation Tension Scale Acute* (DSS-acute; Stiglmayr et al., 2009). The scale consists of one item for depersonalization (feelings of unreality in relation to oneself), somatoform dissociation (sensory and motor disturbances), derealization (feelings of unreality regarding the environment), and analgesia (alterations of sensory processes). The response options ranged from 1 = *not at all* to 4 = *very much*, where higher scores indicated a higher severity of dissociation symptoms (α = 0.85). For the LPA, we calculated the mean score.

#### Frequency of Overt Aggression Toward Peers and Overt Aggression From Peers, Peer Aggression and Peer Victimization

Müller et al. (2012) developed the German Self-Report Behavior Aggression-Opposition Scale, which consists of 12 items with three dimensions (overt aggression, covert aggression, and opposition). To assess aggression toward peers in the classroom as perpetrators and as victims, we used the subscale *overt aggression*, which consists of five items (Müller, 2013): teasing to make angry, physically pushing around, threatening to hurt physically, insulting/offending, and physically hurting. Participants could rate perpetrating resp. being victimized by overt aggression on a 4-point Likert scale: 1 = *never happened*, 2 = *once or twice per month*, 3 = *once per week*, 4 = *more than once per week* since school started, with higher values indicating more frequent perpetration or victimization. As repeated behavior toward another person is often an indicator of peer victimization (Afifi et al., 2020), we adapted Müller’s (2013) response options to determine whether adolescents demonstrated aggression over a long period of time (1 month). For *peer aggression*, the value of Cronbach’s Alpha was [α] = 0.80. For *peer victimization*, the value of Cronbach’s Alpha was [α] = 0.82. For the LPA, we calculated the mean score.

#### Frequency of Class Disruption

Müller et al. (2012) developed the German Fribourg Self- and Peer-Report Scales–School Problem Behavior (FSP-S), which consists of eight items. We adapted Müller’s (2013) response options to determine whether adolescents demonstrated aggression over a long period of time (1 month). Participants could rate class-disruptive behavior on a 4-point Likert scale: ranging from 1 = *never happened*, 2 = *once or twice per month*, 3 = *once per week*, 4 = *more than once per week* (α = 0.82) since school started. Higher values indicated greater frequency of disruptive behavior. We included items such as “Giving the teacher rude answers” and “Not having done your homework.” For the LPA, we calculated the mean score.

### Sociodemographic Covariates

#### Assigned Sex

We omitted sex from class lists that categorized adolescents into males = 0 and females = 1.

#### Migration Background

If the adolescents indicated that they or their parents did not have Swiss nationality or the adolescent him- or herself was not born in Switzerland, then he or she had a migration background (=1). If the above characteristics did not apply, they did not have a migration background (=0).

#### Sociocultural Status

Following Kassis et al., 2013b, we used student sociocultural status as a composite score for students’ socioeconomic background with the dimensions of parental education level, number of books in the household, and education- and computer-related possessions. Because adolescent-reported SES has proven to be complicated, with few knowing about family income or even their parents’ occupation, researchers have recommended the use of multiple indicators with a composite score (Currie et al., 1997; Broer et al., 2019). We gathered information on parental education using the following questions: “What school/education did your mother/father graduate from?” (ranging from 1 = *Elementary school not completed* to 8 = *Higher educational studies/University*) as well as *other* = 9, *don’t know* = 10, *number of own books* on a scale ranging from *0 to 5 books* = 1 to *more than 45 books* = 5, and *number of books in household* on a scale ranging from *0 to 10 books* = 1 to *more than 500 books* = 6 with illustrative pictures, which Hoffmeyer-Zlotnik and Geis (2003) originally developed. We gathered *Financial background* information using the item Educational and computer-related belongings (OECD, 2010) with the question “Which of the objects mentioned below are in your home?” Possible responses were “yes” and “no.” We included the items “A room that is solely yours” and “A computer or tablet you can use for learning.” We developed composite scores from the three scales and divided them into the proficiency ratings *low* = 1, *medium* = 2, and *high* = 3.

### Analysis Plan

Latent profile analysis identifies types or groups of people who exhibit different profiles of personal and/or environmental attributes (Spurk et al., 2020). It is a person-centered analysis very similar to latent class analysis (LCA), but where continuous indicators also can be used rather than categorical ones. The LPA and LCA techniques detect latent subgroups in data by determining the probability that individuals belong to different groups. LCA and LPA are also often referred to as “mixture models.” LPA can be compared to confirmatory factor analysis, except that it extracts latent groups rather than latent constructs. Compared to variable-centered analyses, LPA allows looking closer at profiles and their predictors and distinguishing between subgroups not revealed in the former case (Ferguson et al., 2020). The goal of LPA is to extract distinct and optimally interpretable latent profiles that have a “latent profile model with a high degree of class homogeneity (low within-class variability) along with a high degree of class separation (high between-class variability)” (Masyn, 2013, p. 585).

For all estimations in Mplus version 8.4, we used maximum likelihood estimation with robust standard errors, due to non-normal distributions (Spurk et al., 2020). Further, due to the exploratory nature of the underlying research questions, we did not exclude any cases (Spurk et al., 2020). Missing data was dealt with full information maximum likelihood estimation (FIML). To avoid local solutions, the random starts were raised to 1000 and the final optimizations to 100 (Ferguson et al., 2020). We used the default setting of Mplus to estimate all models, therefore constraining the variance to be equal across classes, but not within classes, and covariances were constrained to 0 (Muthén and Muthén, 2017).

For the first step, we conducted a series of LPAs for the two subsamples “violence” (experiences of serious parental physical violence) and “non-violence” (absence of serious parental physical violence) to assess the accurate number of symptomology profiles for both groups. Akaike’s Information Criterion (AIC); Bayesian Information Criterion (BIC); Sample-Adjusted BIC (aBIC); Entropy; posterior classification probabilities and (adjusted) Lo, Mendell, and Rubin Test (LMR) as well as the bootstrapped likelihood ratio test (BLRT) were available selection criteria. Based on simulation studies, adjusted LMR, BLRT, and BIC are relatively stable selection criteria for the number of profiles regardless of sample size, where Entropy and AIC seem to be unreliable methods for profile number decisions. For most conditions, aBIC performs as well as BLRT except for low (*N* = 250) sample sizes (Tein et al., 2013). In addition, we used the parsimony principle as well as content considerations to finalize the number of profiles (Ferguson et al., 2020). Based on various statistical criteria, we determined which number of profiles most appropriately represented the data for the subsamples at hand (Ferguson et al., 2020). Although we compared all selection criteria, based on the power of the selection criteria and the different sample sizes for youth with violence experiences (*n* = 403) and youth without violence experiences (*n* = 1,571) we focused on adjusted LMR and BIC (Tein et al., 2013). Decreasing BIC, AIC, or aBIC values improved the model. For adjusted LMR and BLRT, a probability value below than.05 indicates that the *K*_0_-profile model fits significantly better to the observed data than a model with a further profile (Tein et al., 2013). To test for mean-level differences of the indicators between the profiles of the respective samples, we conducted pairwise Wald tests.

To test for measurement invariance (MI) on a configural level, we compared the separate LPAs for both samples in terms of the number and shape of the profiles. Configural invariance tests if the same number of profiles is present across groups (Olivera-Aguilar and Rikoon, 2018, p. 441). To test for metric and scalar invariance, the subgroup variable is inserted as the KNOWNCLASS compute in Mplus. Then, unconstrained (free means and variances across the groups), semi-constrained (variances and means fixed across the groups), and fully constrained models (variances and means fixed across and within the groups) are compared through AIC, BIC, and aBIC with lower values indicating the best fitting model. If the semi-constrained model fits better than the unconstrained model, metric invariance holds. If the constrained model fits the data better than the semi-constrained model, scalar invariance holds. If measurement invariance does not hold and therefore, the identified latent profiles have different meanings across groups in terms of typology membership, comparisons across groups are not justified (Olivera-Aguilar and Rikoon, 2018). In most research, MI is neglected and valid comparisons between factor means and/or regression coefficients therefore are potentially biased (Van De Schoot et al., 2015). If MI does not hold, further analysis must be conducted separately across groups (Morin et al., 2016).

In the last step, we applied a three-step approach for auxiliary variables to include class membership predictors of gender, migration background, and sociocultural status. The standard method for including auxiliary variables (predictors) in Mplus is the one-step approach, which has the disadvantage, as Collier and Leite (2017) summarized, that the number of profiles may change when auxiliary variables are included and that the latent variable may lose its significance when the auxiliary variables affect it. A widely acknowledged and recommended method to include auxiliary variables, because it is classification-error corrected (Asparouhov and Muthén, 2014) in Mplus, is the R3STEP command, which was therefore used in the underlying estimations.

## Results

### Descriptive Statistics

Means, standard deviations, and pairwise *t*-tests of the internalizing and externalizing symptom indicators are shown in Table 1. All indicators differed significantly across youths who experienced physical parental violence (from now on referred to as PPV) and youths who did not experience physical parental violence (from now on referred to as NPPV) groups. PPV youth scored higher than NPPV youth in all five indicators. All corrected effect sizes (Hedges *g*; Lakens, 2013) were moderate. The prevalence of physical parental violence was 20.4%.

**TABLE 1:** Descriptive statistics of indicators for PPV and NPPV groups.

Indicator	Mean (*SD*)		CI mean difference	*p*	Hedges *g*
	PPV	NPPV	[LCI, HCI]		
	(*n* = 403)	(*n* = 1571)			
Depression/Anxiety	2.03 (0.69)	1.73 (0.61)	[0.22,0.37]	<0.001	0.48
Dissociation	1.61 (0.74)	1.31 (0.54)	[0.21,0.38]	<0.001	0.51
Peer Aggression	1.58 (0.62)	1.34 (0.48)	[0.19,0.30]	<0.001	0.47
Peer Victimization	1.60 (0.73)	1.33 (0.51)	[0.19,0.34]	<0.001	0.48
Classroom Disruption	1.85 (0.59)	1.66 (0.52)	[0.13,0.26]	<0.001	0.36

*PPV, parental physical violence; NPPV, no parental physical violence; SD, standard deviation; LCI, Lower bound of confidence interval; HCI, higher bound of confidence interval.*

### Single Latent Profiles Separate for Parental Physical Violence and No Parental Physical Violence

For the first step, we conducted a series of single latent profile analyses separately for the adolescents in the PPV and those who were in the NPPV group. For both groups, one to six profiles were evaluated using model fit criteria (see Table 2), focusing on adjusted LMR, and BIC, BLRT profile size, and parsimony principle.

**TABLE 2:** Model fit of the latent profile analysis for PPV and NPPV groups.

Group	No. of latent profiles	AIC	BIC	Adjusted BIC	Entropy	Adjusted LMR LRT *p*	Bootstrap LRT *p*	Smallest profile
PPV (*n* = 403)	1	3878.625	3918.614	3886.883				403 (100%)
	2	3561.835	3625.818	3575.048	0.894	<0.001	<0.001	72 (18%)
	3	3357.795	3445.771	3375.963	0.895	<0.05	<0.001	40 (10%)
	**4**	**3240.640**	**3352.610**	**3263.763**	**0.850**	**<0.05**	**<0.001**	**34** **(8.5%)**
	5	3196.250	3332.213	3224.328	0.874	>0.05	<0.001	11 (2.7%)
	6	3136.570	3296.527	3169.603	0.869	>0.05	<0.001	15 (3.7%)

NPPV (*n* = 1,571)	1	11756.616	11810.210	11778.443				1571 (100%)
	2	10313.349	10399.101	10348.272	0.916	<0.01	<0.001	193 (12.3%)
	3	9320.842	9438.750	9368.861	0.918	<0.01	<0.001	161 (10.2%)
	**4**	**8857.997**	**9008.062**	**8919.112**	**0.906**	**<0.05**	**<0.001**	**82** **(5.2%)**
	5	8486.985	8669.207	8561.197	0.915	<0.01	<0.001	23 (1.5%)
	6	8180.569	8394.948	8267.877	0.911	>0.05	<0.001	23 (1.5%)

*AIC, Akaike Information Criterion; BIC, Bayesian Information Criterion; Adjusted LMR LRT, Lo-Mendell-Rubin adjusted likelihood ratio test; PPV; parental physical violence; NPPV, no parental physical violence. Bold values mean selected number of profiles.*

Starting from the one-profile solution, AIC, BIC, and aBIC values were the highest for both groups, thus indicating the worst fit. For both groups, the two-profile solution decreased AIC, BIC, and aBIC values as well as a significant adjusted LMR and BLTR test, indicating a better fit of the two-profile solution compared to the one-profile solution. Class proportion was similar for both groups with a much higher proportion for one profile compared to the other. Again, for both groups, the AIC, BIC, and aBIC values decreased from the two-profile to the three-profile solution with a minimally higher entropy and significant adjusted LMR and BLTR tests, indicating better fit for the three-profile solution against the two-profile solution. The same is true for the four-profile solution, with lower AIC, BIC, and aBIC values and significant adjusted LMR and BLRT tests, indicating a better fit for the four-profile solution.

For the PPV group, the five-profile solution raised a non-significant adjusted LMR test and a profile with a rather small class proportion (<5%). Taking into account the parsimony principle, the better model fit, and the interpretable profiles in terms of content, a four-profile solution was chosen for the PVV sample. The five-profile solution for the NPPV group had lower IC values as well as a significant adjusted LMR test, but a very small group size with 23 adolescents constituting 1.5% of the sample. For reasons of parsimony, because no further insightful knowledge could be gained with the fifth profile, and because of the rule of thumb that profiles with less than 25 individuals can reduce the accuracy of the profile compared to larger profiles (Spurk et al., 2020), we rejected the five-profile solution for the four-profile solution.

### Latent Profile Descriptions

**FIGURE 1:**
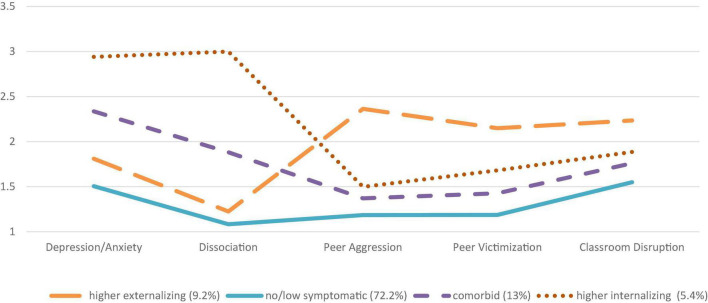
The four profiles of internalizing and externalizing symptoms identified by latent profile analysis (NPPV).

**FIGURE 2:**
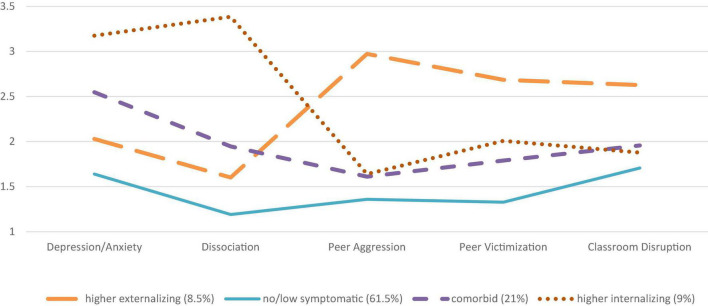
The four profiles of internalizing and externalizing symptoms identified by latent profile analysis (PPV).

Considering both adolescents’ groups (PPV and NPPV) had very similar profiles with respect to the indicators, the profiles in each of the two samples were termed the same (see Figures 1, 2). The first and proportionally biggest profile for both samples was called *no/low symptomatic* because the levels on all five indicators were the lowest with indicator means lower than the respective sample means. Based on our definition of violence resilience, the *no/low symptomatic* profile was resilient to externalizing and internalizing symptomatology. Thus, adolescents in the *no/low symptomatic profile* in the PPV (61.5%) and NPPV (72.2%) groups showed low levels of depression/anxiety symptoms, dissociation, peer aggression, peer victimization, and classroom disruption. The percentage of youth in the *no/low symptomatic* profile was higher in the NPPV group compared to the PPV group.

The second profile was named *comorbid* and was for both groups PPV (21%) and NPPV (13%) composed of above-average levels of the respective samples on depression/anxiety as well as dissociation, peer aggression, peer victimization, and classroom disruption. For both PPV and NPPV, the frequency of peer aggression indicator had the lowest levels on all indicators. The percentage of youths in the *comorbid* profile was higher for the PPV group compared to the NPPV group.

The internalizing indicators (depression/anxiety and dissociation) in the third profile had considerably higher indicator mean levels above the PPV sample means (9%) (see Table 1). The externalizing indicator levels were similar to the *comorbid* profile in the PPV group, with the highest levels for the indicator peer victimization. Therefore, the third profile was called the *higher internalizing* profile, where the PPV group had very high levels on the internalizing indicators, especially dissociation, and still heightened levels on the externalizing indicators as in the *comorbid* profile. In the NPPV group, the *higher internalizing* profile had the fewest individuals (5.4%), making it the smallest profile in the sample without physical parental violence. The mean levels of the indicators for depression/anxiety symptoms as well as dissociation were again considerably high in the NPPV group. The externalizing symptom levels were slightly above the *comorbid* profile.

The fourth profile in the PPV group (8.5%) had considerably higher mean levels in relationship to the indicators for peer aggression and peer victimization as well as classroom disruption. The levels on the internalizing indicators of depression/anxiety as well as dissociation were lower than in the *comorbid* profile, but still higher than in the *no/low symptomatic* profile in the PPV sample. Therefore, the fourth profile was called the *higher externalizing* profile, where the PPV group had very high levels on the externalizing indicators, but had still somewhat heightened levels on the internalizing indicators. In the NPPV group (9.2%), the mean levels of depression/anxiety and dissociation were slightly above the *no/low symptomatic* profile, but lower than the *comorbid* profile. Externalizing indicator levels in the *higher externalizing* profile were the highest for peer aggression, peer victimization, and classroom disruption.

### Measurement Invariance: Testing for Generalizability of the Latent Profiles Across Groups

After we performed the LPA separately for the two groups (PPV and NPPV), the question arose whether the observed profiles could be generalized across the two samples (Olivera-Aguilar and Rikoon, 2018). In other words, whether the observed profiles reflected the same constructs in the subsample with parental physical violence and without. To measure generalizability, we tested measurement invariance in several steps. The first step was done in Section “Single Latent Profiles Separate for Parental Physical Violence and No Parental Physical Violence” by calculating LPAs per group (PPV and NPPV) individually and searching for optimal profile solutions. The selection criteria pointed to a four-profile solution in both subsamples, which corresponds to the configural measurement invariance, meaning that the model form was equivalent for both samples.

The unconstrained model with the four-profile multigroup LPA had a better model fit with lower AIC, BIC, and aBIC values. Further, the LTR test (χ^2^ = 132.48, df = 20, *p* < 0.001) was significant, thus we rejected the equivalent model solutions (see Table 3). In cases of measurement non-invariance, the data can be tested for partial measurement invariance, where some model parameters are restricted to be equal across groups (Kankaraš et al., 2010). Partial measurement invariance did not hold either, as AIC, BIC, and aBIC values were higher for the unconstrained model, and LTR tests were significant. When measurement invariance cannot be established, subsequent analysis and its interpretation must be conducted separately across groups (Morin et al., 2016).

**TABLE 3:** Measurement invariance model comparison.

Model	AIC	BIC	aBIC	Free parameters	H0	H0 scaling factor	X2 (df)	*p*	Result
Unconstrained	14301.861	14592.428	14427.222	52	–7098.931	2.1825	201.64 (23)	<0.001	Rejected
Constrained	14554.787	14716.834	14624.700	29	–7248.394	2.7377			

*AIC, Akaike Information Criterion; BIC, Bayesian Information Criterion; aBIC; sample-adjusted Bayesian Information Criterion; H0, Loglikelihood value.*

### Pairwise Wald Tests for Significant Differences Within the Two Subsamples

To examine whether the profiles differed significantly within each group, we performed pairwise Wald tests. An overall significant Wald test showed that the profiles differed significantly from each other in the PPV group [χ^2^ (15) = 891.429, *p* < 0.001] and NPPV group [χ^2^ (15) = 1132.589, *p* < 0.001]. A pairwise comparison of the indicators can be found in Table 4.

**TABLE 4:** Pairwise comparison of the latent profile indicators separately for PPV and NPPV groups.

Variable	Group	No/low symptomatic^1^ *M* (*SE*)	Comorbid^2^ *M* (*SE*)	Higher Internalizing^3^ *M* (*SE*)	Higher Externalizing^4^ *M* (*SE*)
Depression/Anxiety	PPV	1.639 (0.039) ^2,3,4^	2.547 (0.079)^1,3, 4^	3.715 (0.096) ^1,2,4^	2.029 (0.126) ^1,2,3^
	NPPV	1.505 (0.017) ^2,3,4^	2.336 (0.046)^1,3, 4^	2.940 (0.096)^1,2,4^	1.810 (0.065)^1,2,3^
Dissociation	PPV	1.191 (0.021) ^2,3,4^	1.945 (0.093)^1,3^	3.385 (0.110)^1,2,4^	1.601 (0.152)^1,3^
	NPPV	1.083 (0.007)^2,3,4^	1.882 (0.055)^1,3,4^	2.999 (0.100)^1,2,4^	1.225 (0.036)^1,2,3^
Peer Aggression	PPV	1.360 (0.034)^2,3,4^	1.611 (0.088)^1,4^	1.639 (0.105)^1,4^	2.973 (0.201)^1,2,3^
	NPPV	1.185 (0.012)^2,3,4^	1.371 (0.039)^1,4^	1.498 (0.074)^1,4^	2.364 (0.096)^1,2,3^
Peer Victimization	PPV	1.328 (0.038)^2,3,4^	1.790 (0.152)^1,4^	2.007 (0.190)^1,4^	2.685 (0.250)^1,2,3^
	NPPV	1.187 (0.014)^2,3,4^	1.427(0.055)^1,4^	1.681 (0.128)^1,4^	2.148 (0.094)^1,2,3^
Classroom Disruption	PPV	1.706 (0.042)^2,3,4^	1.957 (0.073)^1,4^	1.878 (0.105)^1,4^	2.627 (0.212)^1,2,3^
	NPPV	1.549 (0.016)^2,3,4^	1.759 (0.045)^1,4^	1.885 (0.075)^1,4^	2.235 (0.068)^1,2,3^

*^1–4^Small numbers indicate significant differences of the pairwise indicator mean levels.*

The *no/low symptomatic* profile mean levels for PPV differed significantly in four out of five symptom indicators from the profiles *comorbid*, *higher internalizing*, and *higher externalizing*. Only the classroom disruption indicator in the *no/low symptomatic* profile did not differ significantly from the *higher internalizing* profile. For the NPPV group, all indicators mean levels in the *no/low symptomatic* profile differed significantly from the other three profiles.

The *comorbid* profile mean levels for PPV differed significantly from the other three profile mean levels only for the depression/anxiety indicator. The dissociation indicator differed significantly in the *comorbid* profile from the *no/low symptomatic* and the *higher internalizing* profile, but not from the *higher externalizing* profile. The indicators of peer aggression, peer victimization, and classroom disruption in the *comorbid* profile differed significantly from the *no/low symptomatic* profile and *higher externalizing* profile, but not from the *higher internalizing* profile. The NPPV group performed the same except for the dissociation indicator, where the mean levels were significantly different from all other profiles.

For the PPV group, mean levels of the *higher internalizing* profile differed significantly from the other three profiles for depression/anxiety as well as dissociation and peer victimization. Mean levels of peer aggression and classroom disruption of the *higher internalizing* profile did not differ significantly from the *comorbid* profile. For the NPPV group, mean levels of peer aggression, classroom disruption, and peer victimization of the *higher internalizing* profile did not differ significantly from the *comorbid* profile.

The mean levels of the symptom indicators of the *higher externalizing* profile differed significantly from all other profiles for the NPPV group. For the PPV group, the mean levels of the symptom indicators differed for all profiles except for dissociation.

Notably, the indicator means for all profiles were higher for the PPV group than for the NPPV group. However, because the measurement invariance between the two subsamples was not given, the mean values of the respective samples should not be compared directly. This is because the profiles – even if visually similar – do not mean the same for the subgroups. Further, the depression/anxiety indicator appears particularly important for distinguishing the four profiles because it significantly distinguishes all profiles.

### Multinomial Logistic Regression (Three-Step Procedure in Mplus) for Sociodemographic Predictor Variables

To assess whether assigned sex, migration background, and sociocultural status predicted profile membership, the automatic three-step procedure of Mplus (R3STEP) was used separately for both subgroups (see Table 5). Assigned sex (1 = female) and migration background (1 = migration background) were dichotomized, and then we inserted sociocultural status as an ordinal variable (low, middle, high).

**TABLE 5:** Three-step multinomial logistic regression analysis with sociodemographic predictors.

	Predictor	Comorbid vs. No/low symptomatic		Higher externalizing vs. No/low symptomatic		Higher internalizing vs. No/low symptomatic		Comorbid vs. Higher internalizing		Higher externalizing vs. Higher internalizing		Comorbid vs. Higher externalizing	
		Estimate (*SE*)	*OR*	Estimate (*SE*)	*OR*	Estimate (*SE*)	*OR*	Estimate (*SE*)	*OR*	Estimate (*SE*)	*OR*	Estimate (*SE*)	*OR*
PPV	Female	1.031** (0.334)	2.803	–1.538* (0.693)	0.215	1.037* (0.402)	2.822	–0.007 (0.487)	0.993	**–2.575** ** (0.773)	0.076	2.569** (0.760)	13.135
	Migration background	0.061 (0.345)	1.063	0.100 (0.476)	1.105	0.111 (0.406)	1.117	–0.049 (0.489)	0.952	–0.011 (0.585)	0.989	–0.0388 (0.561)	1.011
	High sociocultural status	–0.101 (0.283)	0.904	–0.488 (0.359)	0.614	–0.364 (0.323)	0.695	0.263 (0.395)	1.301	–0.125 (0.460)	0.883	0.388 (0.450)	1.133
NPPV	Female	0.451* (180)	1.569	–1.181*** (0.235)	0.307	0.312 (0.249)	1.366	0.139 (0.298)	1.149	–1.493*** (0.328)	0.225	1.632*** (0.280)	5.112
	Migration background	0.060 (0.180)	1.062	0.312 (0.204)	1.366	1.057*** (0.257)	2.877	–0.997** (0.304)	0.369	**–0.745** * (0.314)	0.475	–0.251 (0.255)	0.778
	High sociocultural status	–0.284* (0.136)	0.752	–0.272 (0.154)	0.762	–0.097 (0.180)	0.908	–0.187 (0.220)	0.829	–0.175 (0.228)	0.840	–0.013 (0.195)	0.987

Estimate, β from R3STEP analysis; ***p < 0.001; **p < 0.01; *p < 0.05. Bold values mean significant results.

For the PPV group, females were more likely than males to be in the *comorbid* profile or *higher internalizing* profile than in the *no/low symptomatic* profile. Conversely, females were more likely to be in the *no/low symptomatic* profile, the *higher internalizing* profile, or the *comorbid* profile compared to males. Males were more likely to be in the *higher externalizing* profile than in the *no/low symptomatic* profile compared to females. No other pairwise comparisons in the PPV sample were significant.

For the NPPV group, females were more likely to be in the *comorbid* profile than to be in the *no/low symptomatic* profile or the *higher externalizing* profile compared to males. Vice versa, males were more likely to be in the *higher externalizing* profile than the *no/low symptomatic* profile or the *higher internalizing* profile compared to females. Adolescents with migration background were more likely to be in the *higher internalizing* profile than the *no/low symptomatic* profile. On the other hand, Native adolescents were more likely to be in the *higher internalizing* profile than the *comorbid* profile or the *higher externalizing* profile compared to adolescents with a migration background. Adolescents with lower sociocultural status were more likely to be in the *comorbid* profile than in the *no/low symptomatic* profile.

## Discussion

Based on prior research findings on violence and violence resilience as well as resilience as a theoretical framework, this study aimed to answer the following three questions through LPA and multinomial logistic regression: (1) How many and what kind of non-dichotomous profiles of externalizing and internalizing symptoms related to externalizing and internalizing symptom indicators can we identify using LPA, and how are they composed? (2) Do differences exist in externalizing and internalizing symptom profiles between adolescents with and without experiences of parental physical violence? (3) Do gender, migration background, and sociocultural status predict those profiles?

First, the present study, with a prevalence of parental physical violence of 20.4%, confirmed the tragic international findings: one in five adolescents experience serious physical violence at the hands of their parents (Kapella, 2011; Enzmann et al., 2018; Kassis et al., 2018). Further, we were able to identify four distinct profiles of internalizing and externalizing indicators for youth with and without experiences of parental physical violence. Both groups had a profile termed *no/low symptomatic*, with the largest percentage of youths, and three smaller profiles termed *higher internalizing*, *higher externalizing*, and *comorbid*. Thus, the results confirm our first hypothesis, where we expected four profiles, at least one of which had no/low levels of symptoms and one of which had comorbid symptoms. Contrary to expectations, however, the other two profiles did not clearly emerge as internalizing, externalizing, or clearly comorbid. On the one hand, there was a profile with significantly higher internalizing symptoms, but still with increased externalizing symptoms. And vice versa, a profile with significantly higher externalizing symptoms, but also increased internalizing symptoms. Consequently, the current study identified non-dichotomous psychopathology symptom profiles in the parental physical violence group as well as in the group without having experienced physical family violence. The second hypothesis is confirmed by the fact that the profiles differ between adolescents with and without parental physical violence experiences due to the lack of measurement invariance and thus represent different constructs. Although the profiles were descriptively similar (configural measurement invariant) in the samples with and without violence experiences, the two samples were measurement non-invariant on metric and scalar levels, and thus not comparable on their mean levels. Measurement invariance is a necessary procedure because its lack can seriously misinterpret true mean differences, and non-invariance can even be informative for important conclusions about the differing interpretation of the same construct by different groups (Putnick and Bornstein, 2016). Measurement non-invariance in the present study designates that the indicators measure different latent constructs in the subgroups with and without parental physical violence experiences. Thus, adolescents with physical parental violence experiences may perceive psychopathological symptoms differently than those without physical violence experiences. Similar to findings that show how depression treatment is not as effective for individuals with a history of maltreatment (see Williams et al., 2016), the present findings can be a first step in better understanding psychopathology outcomes regarding violence resilience, as the absence of externalizing and internalizing symptoms. It also demonstrates the importance of focusing on risk-specific resilience. Considering measurement invariance did not hold and the present paper focuses on physical violence experiences, only the group with experiences of physical parental violence will be considered in the next section.

The four symptomatology profiles following parental physical violence show similarities to studies that analyzed person-centered profiles following adversity. In labeling the symptom profiles, the present study was oriented toward existing research. However, the designations must be viewed critically, as all symptom profiles included comorbidity and not solely the *comorbid* profile. Similar to Gallitto et al. (2017), who had a minimal symptom group consisting of 59% of the sample, the present *no/low symptomatic* profile included 61.5% of the sample. Duprey et al.’s (2020) study, which looked at psychopathology profiles changes from early childhood to adolescents following maltreatment, identified a low symptomology profile consisting of 82% of the sample, which had stable low internalizing and externalizing symptoms and was labeled a resilient pathway. Duprey et al. (2020) also found a considerably higher proportion of adolescents with low symptoms, which could be due to different indicators, considering the authors used only one indicator each for externalizing and internalizing symptoms. Berona et al. (2017) found a subclinical group consisting of 43%. Their profiles also consisted of several externalizing and internalizing indicators, but they did not use a random sample. Instead, they studied adolescents who were acutely suicidal and psychiatrically hospitalized, which likely influenced the low group in their symptom levels. In turn, Willner et al. (2016) found a group with internalizing and externalizing indicators described as *well-adjusted* that comprised only 7–11% of the sample, but it included children up to second grade and had an oversample for elevated externalizing symptoms. The current study’s proportions can also be compared to Kassis et al., 2013b study, which found 31% resilient, 28.3% near-resilient, and 40.6% non-resilient youth following parental violence. The present study identified a *no/low symptomatic* profile consisting of 61.5% of the violence sample that included both adolescents with no symptoms and those with relatively low symptoms. This means that the *no/low symptomatic* profile includes both resilient and near-resilient youth, according to Kassis et al., 2013b categorization. As Kassis et al. (2018) showed in their study, even if youth following family violence were considered symptom free regarding depression and aggression, they still showed higher levels in risk factors (e.g., alcohol consumption, drug use) as well as lower levels of protective factors (e.g., self-acceptance, optimism about the future) compared to resilient youth without family violence experiences. Therefore, some caution needs to be taken when considering who to label resilient following physical parental violence and who to label merely symptom-free. Nevertheless, as Luthar et al. (2015) have pointed out, the absence of psychopathology following major risk factors can already be described as resilience. To that end, parental physical violence is a major risk factor (Li et al., 2016; Maneta et al., 2017; Enzmann et al., 2018; Kassis et al., 2018), considering the absence of psychopathological symptoms in adolescents with experiences of developmentally deleterious risk factors is not minor (Luthar et al., 2015). Based on the previous practice of operationalizing violence resilience as the absence of internalizing and externalizing symptoms, this means that adolescents with PPV experiences in the *no/low symptomatic* profile can be considered violence resilient. Further studies should look more closely at indicators that measure healthy development as well. Although not developing psychopathological symptoms despite severe physical parental violence is already a major milestone, future research should examine the extent to which the different profiles vary in terms of healthy development. Because resilience can only ever emerge in the context of adversity, it is particularly important to consider risk factors when studying resilience and to incorporate them into decisions about what criteria must be met for resilient development.

Willner et al. (2016) summarized studies indicating that children with internalizing–externalizing comorbidity tend to exhibit the most severe symptoms and that many studies nevertheless often analyzed the two symptom categories separately. In their study, Willner et al. (2016) found a comorbid group consisting of 48% of the sample with the highest externalizing and internalizing symptoms. Berona et al. (2017) analyzed 433 acutely suicidal, psychiatrically hospitalized adolescents and found two comorbid profiles with moderate (17.1%) and severe (10.9%) levels of the anxious/depressed, attention problems, and aggressive behavior indicators. Duprey et al. (2020) found a high comorbidity class of 6.43% of the sample who had persistent high externalizing and internalizing symptoms over time and a higher risk of suicidal ideation, suicide plan, and suicide attempt. In the present study, although cross-sectional, the *comorbid* profile (21%) also consisted of youth with elevated levels of externalizing and internalizing symptoms, similar to Berona et al.’s (2017) moderate profile. However, the *comorbid* profile included a different proportion of adolescents than the high comorbidity classes in the Willner et al.’s (2016) and Duprey et al.’s (2020) studies, and symptom levels were considerably lower compared to the *higher internalizing* and *higher externalizing* profiles. This may be due to the fact that Duprey et al.’s (2020) study had a larger sample of adolescents with experiences of violence or other risk factors; thus, the present sample may have been too small to find more refined subcategories of adolescents with very high internalizing and externalizing symptoms. However, it is also possible that this profile is evident due to Duprey et al.’s (2020) criteria-based and non-random sample and is less evident in a random sample. On the other hand, Willner et al. (2016) oversampled children with externalizing symptoms, which might explain their high percentage. They concluded that a common vulnerability factor contributed to the stable comorbidity of internalizing and externalizing symptoms and some children with externalizing symptoms are at risk of later developing internalizing symptoms (Willner et al., 2016). This could also explain the different results, considering the present sample consisted of adolescents and therefore those externalizing symptoms possibly developed into internalizing symptoms with age. Riina et al.’s (2014) finding could also support this because they found that externalizing symptoms in individuals with experiences of parental violence manifest predominantly at a younger age and decrease over time. In contrast, internalizing symptoms increase and are particularly pronounced in adolescence. Importantly, future studies should explore how the trajectories of the comorbid profile and the no/low symptomatic profile of the present study change over time and whether the internalizing and externalizing symptoms increase or decrease. Additionally, to identify particularly vulnerable youth, it would also be important to consider suicide-related outcomes as suicidal ideation.

Another indication of the importance of accounting for comorbidity comes from the pairwise Wald tests. The depression/anxiety indicator appeared to be particularly important to distinguish the four profiles, as all pairwise comparisons of all indicators were significant for all four profiles. This means that adolescents in the four profiles differed significantly from each other on all mean levels. Dissociation as an indicator did not appear to differ significantly between the *comorbid* and *higher externalizing* profiles, and the mean levels of all three externalizing indicators did not differ significantly between youths in the *comorbid* and *higher internalizing* profiles. Thus, the question arises whether the four profiles emerged mainly because of the Depression/Anxiety indicator and otherwise less distinct profiles with higher internalizing and externalizing indicators would have emerged.

In the present study, the *higher internalizing* profile accounts for 9% of the group with physical parental violence experiences and the *higher externalizing* profile consists of 8.5%. Thus, the present study confirms the existence of two distinct profiles, with either highly elevated scores for internalizing (Willner et al., 2016; Berona et al., 2017) or externalizing symptoms (Willner et al., 2016; Duprey et al., 2020), and somewhat elevated scores for either externalizing or internalizing symptoms. With respect to the *externalizing profile*, Willner et al. (2016) noted that, “several studies failed to identify a group of children exhibiting high levels of externalizing symptoms only. This profile again highlights the value of examining the full spectrum of symptom expression rather than relying on arbitrary thresholds” (p. 5). They demonstrated that person-centered consideration of symptoms could reveal individuals who might be classified as externalizing only, but in reality exhibit significant elevations of internalizing symptoms (Willner et al., 2016). In this context, the question arises as to how the protective factors for the development of violence resilience that have been demonstrated to date relate to the different profiles.

The performed multinomial logistic regression analysis showed that in the group with experiences of parental physical violence, neither migration background nor sociocultural status predicted the profile to which an individual belonged. Therefore, the third hypothesis, which stated that with low SES and migration background the chance of being in the resilient profile decreases, must be rejected. These findings confirm the results of Belhadj Kouider et al.’s (2014) review study, which showed there were no major differences for youth with migration background and Native youth concerning externalizing symptoms. The findings are also similar to Kassis et al., 2013b, who did not find any differences for migration background and resilience outcomes, though it differs because they found small differences for high SES. Contrary to Duprey et al. (2020), gender differences exist in the present study. Females rather than males have an increased likelihood to be in the *comorbid* or *higher internalizing* profiles in comparison to the *no/low symptomatic* profile. Conversely, females rather than males have an increased likelihood to be in the *no/low symptomatic*, *higher internalizing*, and *comorbid* profiles in comparison to the *higher externalizing* profile. As Yule et al. (2019) noted, diversity issues have been under-researched to date in relation to violence resilience. Although the study, in comparison to other studies, took into account individual characteristics, recent studies exist that reveal how the categorization of sociodemographic variables in current research falls short. As Kassis et al. (2021) have recently shown, thanks to person-centered gender identity and sexual attraction classes, psychosocial status can be mapped in a far more heterogeneous and detailed manner when multidimensional gender identity is considered rather than assigned sex. A more intersectional analysis of youths who have experienced violence would be another way for future research to fully explore the issue.

## Limitations

The present study identified important nuances in distinguishing subgroups of psychopathology symptoms in youth following parental physical violence in a violence resilience framework. Nevertheless, there are some limitations. First, although much of the violence resilience research identifies the absence or low levels of psychopathological symptoms in adolescents as violence resilience, this perspective shows only one side of the coin. From the present findings, it is not possible to infer whether the adolescents in the *no/low symptomatic* profile have developed healthily, but only that they do not exhibit any prominent internalizing or externalizing symptoms. Second, we only considered serious parental physical violence in a dichotomous way without considering less severe forms or varying frequencies of physical violence as well as other types of violence. This is because, although the existing sample of nearly 2,000 youth was relatively large, the person-centered method with additional maltreatment subgroups would have resulted in smaller subsamples for which the chosen method would not have yielded qualitative results. As the number of indicators and the sample size influence the information value of the latent profiles (Spurk et al., 2020). Third, although the study included certain sociodemographic variables, these were dichotomized. Because resilience is a highly contextual construct, verifying the present profiles is necessary with samples across other countries, ages, cultures, and other contexts, such as outside of school. Fourth, the adolescents’ self-report data may contain biases due to social desirability. Further studies should consider using multi-perspective data, such as parent- and teacher-reported information. Lastly, the results are from a cross-sectional study, thus, we cannot draw conclusions about causality. In view of the processual nature of resilience, it would be desirable for future research to examine the profiles longitudinally.

## Conclusion

The present study demonstrated, that contrary to prevailing research, comorbid internalizing and externalizing symptoms were found to different extents in three out of four profiles. Although the *higher externalizing* profile had significantly higher externalizing symptom levels, the levels on the internalizing symptoms were significantly above the *no/low symptomatic* profile. The same was true for the profile *higher internalizing* and in the profile *comorbid* the levels of both symptom groups were elevated. We were able to show that internalizing and externalizing symptoms go hand in hand, albeit to different degrees. Adolescents who are particularly noticeable at school due to externalizing symptoms, should therefore not be stereotyped, but also supported with regard to internalizing symptoms, and vice versa. From an empirical perspective, considering symptom profiles and thus a non-dichotomous operationalization of violence resilience can be a promising approach. Protective factors and additional risk factors can be taken into account in a more targeted way, and thus more specific measures for promoting resilience pathways for adolescents can be developed. Another important finding is that migration background is not associated with increased externalizing symptomatology when internalizing and externalizing symptomatology are not considered separately.

## Data Availability Statement

The raw data supporting the conclusions of this article will be made available by the authors, without undue reservation.

## Ethics Statement

The studies involving human participants were reviewed and approved by Research Ethics Committee, University of Zurich, Switzerland. Written informed consent to participate in this study was provided by the participants’ legal guardian/next of kin.

## Author Contributions

DA collected the data, conducted the analysis, planned, and wrote the manuscript. CF collected the data, contributed to the planning, layout, methodological section and revision of the manuscript. CJ contributed to the manuscript layout and revision as well as to the statistical sections of the manuscript. BE contributed to the manuscript revision and the methodological section. All authors contributed to the article and approved the submitted version.

## Conflict of Interest

The authors declare that the research was conducted in the absence of any commercial or financial relationships that could be construed as a potential conflict of interest.

## Publisher’s Note

All claims expressed in this article are solely those of the authors and do not necessarily represent those of their affiliated organizations, or those of the publisher, the editors and the reviewers. Any product that may be evaluated in this article, or claim that may be made by its manufacturer, is not guaranteed or endorsed by the publisher.

## References

[B1] Afifi T. O. MacMillan H. L. (2011). Resilience following child maltreatment: A review of protective factors. *Can. J. Psychiatr.* 56 266–272. 10.1177/070674371105600505 21586192

[B2] Afifi T. O. Taillieu T. Salmon S. Davila I. G. Stewart-Tufescu A. Fortier J. (2020). Adverse childhood experiences (ACEs), peer victimization, and substance use among adolescents. *Child Abuse Negl.* 106:104504. 10.1016/j.chiabu.2020.104504 32402816

[B3] Arslan G. (2016). Psychological maltreatment, emotional and behavioral problems in adolescents: The mediating role of resilience and self-esteem. *Child Abuse Negl.* 52 200–209. 10.1016/j.chiabu.2015.09.010 26518981

[B4] Asgeirsdottir B. B. Gudjonsson G. H. Sigurdsson J. F. Sigfusdottir I. D. (2010). Protective processes for depressed mood and anger among sexually abused adolescents: The importance of self-esteem. *Personal. Individ. Diff.* 49 402–407. 10.1177/1079063216667917 27591752

[B5] Asparouhov T. Muthén B. (2014). Auxiliary Variables in Mixture Modeling: Three-Step Approaches Using Mplus. *Struct. Equat. Model. Multidiscipl. J.* 21 329–341. 10.1080/10705511.2014.915181

[B6] Baydar N. Akcinar B. (2018). Reciprocal relations between the trajectories of mothers’ harsh discipline, responsiveness and aggression in early childhood. *J. Abnorm. Child Psychol.* 46 83–97. 10.1007/s10802-017-0280-y 28215022

[B7] Belhadj Kouider E. Koglin U. Petermann F. (2014). Emotional and behavioral problems in migrant children and adolescents in Europe: A systematic review. *Euro. Child Adolesc. Psychiatr.* 23 373–391. 10.1007/s00787-013-0485-8 24132833

[B8] Benedini K. M. Fagan A. A. Gibson C. L. (2016). The cycle of victimization: The relationship between childhood maltreatment and adolescent peer victimization. *Child Abuse Negl.* 59 111–121. 10.1016/j.chiabu.2016.08.003 27568065

[B9] Berona J. Horwitz A. G. Czyz E. K. King C. A. (2017). Psychopathology profiles of acutely suicidal adolescents: Associations with post-discharge suicide attempts and rehospitalization. *J. Affec. Disord.* 209 97–104. 10.1016/j.jad.2016.10.036 PMC5473155 27894037

[B10] Bottrell D. (2009). Understanding ‘Marginal’ Perspectives: Towards a Social Theory of Resilience. *Qual. Soc. Work* 8 321–339. 10.1177/1473325009337840

[B11] Bowen N. K. Lee J. Weller B. E. (2007). Social environmental risk and protection: a typology with implications for elementary schools. *Child. Sch.* 29 229–242. 10.1093/cs/29.4.229 21709821 PMC3119897

[B12] Broer M. Bai Y. Fonseca F. (2019). “A review of the literature on socioeconomic status and educational achievement,” in *Socioeconomic Inequality and Educational Outcomes. IEA Research for Education*, Vol. 5. (Cham: Springer). 10.1007/978-3-030-11991-1_2

[B13] Chapman D. P. Whitfield C. L. Felitti V. J. Dube S. R. Edwards V. J. Anda R. F. (2004). Adverse childhood experiences and the risk of depressive disorders in adulthood. *J. Affec. Disord.* 82 217–225. 10.1016/j.jad.2003.12.013 15488250

[B14] Cicchetti D. Rogosch F. A. (2012). Gene × Environment interaction and resilience: Effects of child maltreatment and serotonin, corticotropin releasing hormone, dopamine, and oxytocin genes. *Dev. Psychopathol.* 24 411–427. 10.1017/S0954579412000077 22559122 PMC3684053

[B15] Cicchetti D. Toth S. L. (2015). *Child Maltreatment.* In *Handbook of Child Psychology and Developmental Science: Socioemotional Processes, Vol. 3, 7th ed.* New Jersey: John Wiley & Sons, 513–563. 10.1002/9781118963418.childpsy313

[B16] Collier Z. K. Leite W. L. (2017). A comparison of three-step approaches for auxiliary variables in latent class and latent profile analysis. *Struct. Equat. Model. Multidiscipl. J.* 24 819–830. 10.1080/10705511.2017.1365304

[B17] Collishaw S. Pickles A. Messer J. Rutter M. Shearer C. Maughan B. (2007). Resilience to adult psychopathology following childhood maltreatment: Evidence from a community sample. *Child Abuse Negl.* 31 211–229. 10.1016/j.chiabu.2007.02.004 17399786

[B18] Colizzi M. Costa R. Todarello O. (2015). Dissociative symptoms in individuals with gender dysphoria: is the elevated prevalence real? *Psychiatry Res.* 226 173–180. 10.1016/j.psychres.2014.12.045 25656174

[B19] Currie C. Elton R. Todd J. Platt S. (1997). Indicators of socioeconomic status for adolescents: The WHO Health Behaviour in School-Aged Children Survey. *Health Educ. Res.* 12 385–397. 10.1093/her/12.3.385 10174221

[B20] Dang M. T. (2014). Social connectedness and self-esteem: Predictors of resilience in mental health among maltreated homeless youth. *Iss. Ment. Health Nurs.* 35 212–219. 10.3109/01612840.2013.860647 24597587

[B21] Davis J. P. Ingram K. M. Merrin G. J. Espelage D. L. (2020). Exposure to parental and community violence and the relationship to bullying perpetration and victimization among early adolescents: A parallel process growth mixture latent transition analysis. *Scand. J. Psychol.* 61 77–89. 10.1111/sjop.12493 30278116

[B22] Derogatis L. R. Lipman R. S. Rickels K. Uhlenhuth E. H. Covi L. (1974). The Hopkins Symptom Checklist (HSCL): A measure of primary symptom dimensions. *Mod. Probl. Pharmacopsychiatr.* 7 79–110. 10.1159/000395070 4607278

[B23] DuMont K. A. Widom C. S. Czaja S. J. (2007). Predictors of resilience in abused and neglected children grown-up: The role of individual and neighborhood characteristics. *Child Abuse Negl.* 31 255–274. 10.1016/j.chiabu.2005.11.015 17386940

[B24] Duprey E. B. Oshri A. Liu S. (2020). Developmental pathways from child maltreatment to adolescent suicide-related behaviors: The internalizing and externalizing comorbidity hypothesis. *Dev. Psychopathol.* 32 945–959. 10.1017/S0954579419000919 31407646 PMC7306177

[B25] Enzmann D. Kivivuori J. Marshall I. H. Steketee M. Hough M. Killias M. (2018). *A Global Perspective on Young People as Offenders and Victims.* Cham: Springer.

[B26] Evans S. E. Davies C. DiLillo D. (2008). Exposure to domestic violence: A meta-analysis of child and adolescent outcomes. *Aggress. Viol. Behav.* 13 131–140. 10.1016/j.avb.2008.02.005

[B27] Federal Statistical Office. (2020). *Population by migration status*. Federal Statistical Office. Availabe online at https://www.bfs.admin.ch/bfs/en/home/statistics/population/migration-integration/by-migration-status.html

[B28] Ferguson S. L. Moore G. Whitney E. Darrel M. (2020). Finding latent groups in observed data: A primer on latent profile analysis in Mplus for applied researchers. *Int. J. Behav. Dev.* 44 458–468. 10.1177/0165025419881721

[B29] Flores E. Cicchetti D. Rogosch F. A. (2005). Predictors of resilience in maltreated and nonmaltreated Latino children. *Dev. Psychol.* 41:338. 10.1037/0012-1649.41.2.338 15769190

[B30] Frick P. (1991). Alabama Parenting Questionnaire. *Univers. Alab. Commun. Stud.* 48 59–75.

[B31] Gallitto E. Lyons J. Weegar K. Romano E. (2017). Trauma-symptom profiles of adolescents in child welfare. *Child Abuse Negl.* 68 25–35. 10.1016/j.chiabu.2017.03.011 28391075

[B32] Garmezy N. (1991). Resilience in children’s adaptation to negative life events and stressed environments. *Pediatr. Ann.* 20 459–466. 10.3928/0090-4481-19910901-05 1945543

[B33] Gerin M. I. Hanson E. Viding E. McCrory E. J. (2019). A review of childhood maltreatment, latent vulnerability and the brain: Implications for clinical practice and prevention. *Adopt. Foster.* 43 310–328. 10.1177/0308575919865356

[B34] Go M. Chu C. M. Barlas J. Chng G. S. (2017). The role of strengths in anger and conduct problems in maltreated adolescents. *Child Abuse Negl.* 67 22–31. 10.1016/j.chiabu.2017.01.028 28242364

[B35] Guo L. Wang W. Li W. Zhao M. Wu R. Lu C. (2021). Childhood maltreatment predicts subsequent anxiety symptoms among Chinese adolescents: The role of the tendency of coping styles. *Transl. Psychiatr.* 11 1–10. 10.1038/s41398-021-01463-y 34078876 PMC8172629

[B36] Hazen A. L. Connelly C. D. Roesch S. C. Hough R. L. Landsverk J. A. (2009). Child Maltreatment Profiles and Adjustment Problems in High-Risk Adolescents. *J. Interpers. Viol.* 24 361–378. 10.1177/0886260508316476 18391059

[B37] Hoffmeyer-Zlotnik J. H. Geis A. J. (2003). Berufsklassifikation und Messung des beruflichen Status/Prestige. *ZUMA Nachrichten* 27 125–138.

[B38] Hymel S. Swearer S. M. (2015). Four decades of research on school bullying: An introduction. *Am. Psychol.* 70:293. 10.1037/a0038928 25961310

[B39] Kankaraš M. Moors G. Vermunt J. K. (2010). Testing for measurement invariance with latent class analysis. *Int. Sociol.* 24 1–23.

[B40] Kapella O. (2011). *Gewalt in der Familie und im nahen sozialen Umfeld: Österreichische Prävalenzstudie zur Gewalt an Frauen und Männern.* Vienna: Österr. Inst. für Familienforschung an der Univ.

[B41] Kassis W. Aksoy D. Favre C. A. Artz S. T.-G. (2021). Multidimensional and intersectional gender identity and sexual attraction patterns of adolescents for quantitative research. *Front. Psychol.* 12:697373. 10.3389/fpsyg.2021.697373 34603126 PMC8485041

[B42] Kassis W. Artz S. Maurovic I. Simões C. (2018). What doesn’t kill them doesn’t make them stronger: Questioning our current notions of resilience. *Child Abuse Negl.* 78 71–84. 10.1016/j.chiabu.2017.12.011 29254696

[B43] Kassis W. Artz S. Moldenhauer S. (2013a). Laying Down the Family Burden: A Cross-Cultural Analysis of Resilience in the Midst of Family Violence. *Child Youth Serv.* 34 37–63. 10.1080/0145935X.2013.766067

[B44] Kassis W. Artz S. Moldenhauser S. Geczey I. Rossiter K. (2015). A Dynamic and Gender Sensitive Understanding of Adolescents’ Personal and School Resilience Characteristics. *Int. J. Child Youth Fam. Stud.* 6 388–420. 10.18357/ijcyfs.63201513562

[B45] Kassis W. Artz S. Scambor C. Scambor E. Moldenhauer S. (2013b). Finding the way out: A non-dichotomous understanding of violence and depression resilience of adolescents who are exposed to family violence. *Spec. Iss. Risk Resilie. Contex. Child Maltreat.* 37 181–199. 10.1016/j.chiabu.2012.11.001 23260117

[B46] Lakens D. (2013). Calculating and reporting effect sizes to facilitate cumulative science: A practical primer for t-tests and ANOVAs. *Front. Psychol.* 4:863. 10.3389/fpsyg.2013.00863 24324449 PMC3840331

[B47] Lanier P. Maguire-Jack K. Lombardi B. Frey J. Rose R. A. (2018). Adverse childhood experiences and child health outcomes: Comparing cumulative risk and latent class approaches. *Mater. Child Health J.* 22 288–297. 10.1007/s10995-017-2365-1 28929420

[B48] Li M. D’arcy C. Meng X. (2016). Maltreatment in childhood substantially increases the risk of adult depression and anxiety in prospective cohort studies: Systematic review, meta-analysis, and proportional attributable fractions. *Psychol. Med.* 46 717–730. 10.1017/S0033291715002743 26708271

[B49] Luthar S. S. Brown P. J. (2007). Maximizing resilience through diverse levels of inquiry: Prevailing paradigms, possibilities, and priorities for the future. *Dev. Psychopathol.* 19 931–955. 10.1017/S0954579407000454 17705909 PMC2190297

[B50] Luthar S. S. Cicchetti D. Becker B. (2000). The construct of resilience: A critical evaluation and guidelines for future work. *Child Dev.* 71 543–562. 10.1111/1467-8624.00164 10953923 PMC1885202

[B51] Luthar S. S. Grossman E. J. Small P. J. (2015). “Resilience and adversity,” in *Handbook of Child Psychology and Developmental Science: Socioemotional Processes*, eds Lamb M. E. Lerner R. M. (Hoboken: John Wiley & Sons, Inc), 247–286.

[B52] Macfie J. Cicchetti D. Toth S. L. (2001). Dissociation in maltreated versus nonmaltreated preschool-aged children. *Child Abuse Negl.* 25 1253–1267. 10.1016/s0145-2134(01)00266-6 11700697

[B53] Maneta E. K. White M. Mezzacappa E. (2017). Parent-child aggression, adult-partner violence, and child outcomes: A prospective, population-based study. *Child Abuse Negl.* 68 1–10. 10.1016/j.chiabu.2017.03.017 28388466

[B54] Mariscal E. S. (2020). Resilience following exposure to intimate partner violence and other violence: A comparison of Latino and non-Latino youth. *Children Youth Serv. Rev.* 113:104975. 10.1016/j.childyouth.2020.104975

[B55] Masten A. S. (2001). Ordinary magic: Resilience processes in development. *Am. Psychol.* 56:227. 10.1037//0003-066x.56.3.227 11315249

[B56] Masten A. S. (2014). Global perspectives on resilience in children and youth. *Child Dev.* 85 6–20. 10.1111/cdev.12205 24341286

[B57] Masten A. S. (2019). Resilience from a developmental systems perspective. *World Psychiatr.* 18:101. 10.1002/wps.20591 30600628 PMC6313232

[B58] Masten A. S. Barnes A. J. (2018). Resilience in children: Developmental perspectives. *Children* 5:98. 10.3390/children5070098 30018217 PMC6069421

[B59] Masyn K. E. (2013). Latent class analysis and finite mixture modeling. *Oxford Handb. Quantit. Methods* 2:551.

[B60] McDonald S. E. Corona R. Maternick A. Ascione F. R. Williams J. H. Graham-Bermann S. A. (2016a). Children’s exposure to intimate partner violence and their social, school, and activities competence: Latent profiles and correlates. *J. Fam. Viol.* 31 849–864. 10.1007/s10896-016-9846-7

[B61] McDonald S. E. Graham-Bermann S. A. Maternick A. Ascione F. R. Williams J. H. (2016b). Patterns of adjustment among children exposed to intimate partner violence: A person-centered approach. *J. Child Adolesc. Trauma* 9 137–152. 10.1007/s40653-016-0079-y

[B62] Meng X. Fleury M.-J. Xiang Y.-T. Li M. D’arcy C. (2018). Resilience and protective factors among people with a history of child maltreatment: A systematic review. *Soc. Psychiatr. Psychiatr. Epidemiol.* 53 453–475. 10.1007/s00127-018-1485-2 29349479

[B63] Moody G. Cannings-John R. Hood K. Kemp A. Robling M. (2018). Establishing the international prevalence of self-reported child maltreatment: A systematic review by maltreatment type and gender. *BMC Public Health* 18:1164. 10.1186/s12889-018-6044-y 30305071 PMC6180456

[B64] Morin A. J. S. Meyer J. P. Creusier J. Biétry F. (2016). Multiple-Group Analysis of Similarity in Latent Profile Solutions. *Organ. Res. Methods* 19 231–254. 10.1177/1094428115621148

[B65] Müller C. (2013). Dissoziale Verhaltensweisen und Einstellungen im Längsschnitt erfassen—Entwicklung und Evaluation der „Freiburger Selbst- und Peerauskunftsskalen—Dissozialität“. Heilpädagogische Forschung*. 2–13. (accessed October 31, 2021).

[B66] Müller C. Begert T. Huber C. Gmünder L. (2012). Die “Freiburger Selbst- und Peerauskunftsskalen- Schulisches Problemverhalten”- Entwicklungen und Evaluation eines Verfahrens zur Verlaufsmessung von unterrichtsbezogenen Verhaltensproblemen. *Empiris. Sonderpädagogik* 4 3–21.

[B67] Muthén L. K. Muthén B. O. (2017). *Mplus User’s Guide*, 8th Edn. Los Angeles, CA: Muthén & Muthén.

[B68] Nelon J. L. De Pedro K. T. Gilreath T. D. Patterson M. S. Holden C. B. Esquivel C. H. (2019). A latent class analysis of the co-occurrence of sexual violence, substance use, and mental health in youth. *Subs. Use Misuse* 54 1938–1944. 10.1080/10826084.2019.1618337 31131676

[B69] Nishimi K. Choi K. W. Cerutti J. Powers A. Bradley B. Dunn E. C. (2020). Measures of adult psychological resilience following early-life adversity: How congruent are different measures? *Psychol. Med.* 51 2637–2646. 10.1017/S0033291720001191 32406816 PMC7863576

[B70] Norman R. E. Byambaa M. De R. Butchart A. Scott J. Vos T. (2012). The long-term health consequences of child physical abuse, emotional abuse, and neglect: A systematic review and meta-analysis. *PLoS Med.* 9:e1001349. 10.1371/journal.pmed.1001349 23209385 PMC3507962

[B71] OECD. (2010). *PISA 2009 Ergebnisse.* OECD Library, 10.1787/9789264095335-de (accessed September 25, 2021).

[B72] Olivera-Aguilar M. Rikoon S. H. (2018). Assessing Measurement Invariance in Multiple-Group Latent Profile Analysis. *Struct. Equat. Model. Multidiscipl. J.* 25 439–452. 10.1080/10705511.2017.1408015

[B73] Parra G. R. DuBois D. L. Sher K. J. (2006). Investigation of profiles of risk factors for adolescent psychopathology: A person-centered approach. *J. Clin. Child Adolesc. Psychol.* 35 386–402. 10.1207/s15374424jccp3503_4 16836476

[B74] Putnick D. L. Bornstein M. H. (2016). Measurement invariance conventions and reporting: The state of the art and future directions for psychological research. *Dev. Rev.* 41 71–90. 10.1016/j.dr.2016.06.004 27942093 PMC5145197

[B75] Rebbe R. Nurius P. S. Ahrens K. R. Courtney M. E. (2017). Adverse childhood experiences among youth aging out of foster care: A latent class analysis. *Children Youth Serv. Rev.* 74 108–116. 10.1016/j.childyouth.2017.02.004 28458409 PMC5404688

[B76] Rehan W. Antfolk J. Johansson A. Jern P. Santtila P. (2017). Experiences of severe childhood maltreatment, depression, anxiety and alcohol abuse among adults in Finland. *PLoS One* 12:e0177252. 10.1371/journal.pone.0177252 28481912 PMC5421798

[B77] Riina E. M. Martin A. Brooks-Gunn J. (2014). Parent-to-child physical aggression, neighborhood cohesion, and development of children’s internalizing and externalizing. *J. Appl. Dev. Psychol.* 35 468–477. 10.1016/j.appdev.2014.04.005 PMC12382824 40881355

[B78] Rivera P. M. Fincham F. D. Bray B. C. (2018). Latent Classes of Maltreatment: A Systematic Review and Critique. *Child Maltreat.* 23 3–24. 10.1177/1077559517728125 28875728 PMC5614894

[B79] Runyon M. K. Deblinger E. Steer R. A. (2014). PTSD symptom cluster profiles of youth who have experienced sexual or physical abuse. *Child Abuse Negl.* 38 84–90. 10.1016/j.chiabu.2013.08.015 24148275

[B80] Rutter M. (1993). Resilience: Some conceptual considerations. *J. Adolesc. Health* 14 626–631. 10.1016/1054-139x(93)90196-v 8130234

[B81] Rutter M. (2013). Annual research review: Resilience–clinical implications. *J. Child Psychol. Psychiatr.* 54 474–487. 10.1111/j.1469-7610.2012.02615.x 23017036

[B82] Sexton M. B. Hamilton L. McGinnis E. W. Rosenblum K. L. Muzik M. (2015). The roles of resilience and childhood trauma history: Main and moderating effects on postpartum maternal mental health and functioning. *J. Affect. Disord.* 174 562–568. 10.1016/j.jad.2014.12.036 25560192 PMC4339466

[B83] Shields A. Cicchetti D. (2001). Parental maltreatment and emotion dysregulation as risk factors for bullying and victimization in middle childhood. *J. Clin. Child Psychol.* 30 349–363. 10.1207/S15374424JCCP3003_7 11501252

[B84] Spurk D. Hirschi A. Wang M. Valero D. Kauffeld S. (2020). Latent profile analysis: A review and “how to” guide of its application within vocational behavior research. *J. Vocat. Behav.* 120:103445. 10.1016/j.jvb.2020.103445

[B85] Stiglmayr C. Schmahl C. Bremner J. D. Bohus M. Ebner-Priemer U. (2009). Development and psychometric characteristics of the DSS-4 as a short instrument to assess dissociative experience during neuropsychological experiments. *Psychopathology* 42 370–374. 10.1159/000236908 19752590

[B86] Straus M. A. Gelles R. J. Stienmetz S. K. (2017). *Behind Closed doors: Violence in the American Family.* Milton Park: Routledge.

[B87] Tein J.-Y. Coxe S. Cham H. (2013). Statistical Power to Detect the Correct Number of Classes in Latent Profile Analysis. *Struct. Equat. Model. ?Multidiscipl. J.* 20 640–657. 10.1080/10705511.2013.824781 PMC3904803 24489457

[B88] Teisl M. Cicchetti D. (2008). Physical abuse, cognitive and emotional processes, and aggressive/disruptive behavior problems. *Soc. Dev.* 17 1–23.

[B89] Tlapek S. M. Auslander W. Edmond T. Gerke D. Schrag R. V. Threlfall J. (2017). The moderating role of resiliency on the negative effects of childhood abuse for adolescent girls involved in child welfare. *Children Youth Serv. Rev.* 73 437–444. 10.1186/s12913-016-1423-5 27409075 PMC4943498

[B90] Tschoeke S. Bichescu-Burian D. Steinert T. Flammer E. (2021). History of childhood trauma and association with borderline and dissociative features. *.J. Nerv. Ment. Dis.* 209 137–143. 10.1097/NMD.0000000000001270 33208712

[B91] Ungar M. (2004). A constructionist discourse on resilience: Multiple contexts, multiple realities among at-risk children and youth. *Youth Soc.* 35 341–365. 10.1177/0044118x03257030

[B92] Ungar M. Liebenberg L. Dudding P. Armstrong M. Van de Vijver F. J. (2013). Patterns of service use, individual and contextual risk factors, and resilience among adolescents using multiple psychosocial services. *Child Abuse Negl.* 37 150–159. 10.1016/j.chiabu.2012.05.007 23260119

[B93] Van De Schoot R. Schmidt P. De Beuckelaer A. Lek K. Zondervan-Zwijnenburg M. (2015). Editorial: Measurement Invariance. *Front. Psychol.* 6:1064. 10.3389/fpsyg.2015.01064 26283995 PMC4516821

[B94] Vanderbilt-Adriance E. Shaw D. S. (2008). Conceptualizing and re-evaluating resilience across levels of risk, time, and domains of competence. *Clin. Child Fam. Psychol. Rev.* 11:30. 10.1007/s10567-008-0031-2 18379875 PMC2683037

[B95] Walsh K. Fortier M. A. DiLillo D. (2010). Adult coping with childhood sexual abuse: A theoretical and empirical review. *Aggress. Viol. Behav.* 15 1–13. 10.1016/j.avb.2009.06.009 20161502 PMC2796830

[B96] Wiggins J. L. Mitchell C. Hyde L. W. Monk C. S. (2015). Identifying early pathways of risk and resilience: The codevelopment of internalizing and externalizing symptoms and the role of harsh parenting. *Dev. Psychopathol.* 27 1295–1312. 10.1017/S0954579414001412 26439075 PMC4961476

[B97] Williams L. M. Debattista C. Duchemin A. M. Schatzberg A. F. Nemeroff C. B. (2016). Childhood trauma predicts antidepressant response in adults with major depression: data from the randomized international study to predict optimized treatment for depression. *Transl. Psychiatry* 6:e799. 10.1038/tp.2016.61 27138798 PMC5070060

[B98] Willner C. J. Gatzke-Kopp L. M. Bray B. C. (2016). The dynamics of internalizing and externalizing comorbidity across the early school years. *Dev. Psychopathol.* 28 1033–1052. 10.1017/S0954579416000687 27739391 PMC5319409

[B99] Wright E. M. Fagan A. A. Pinchevsky G. M. (2013). The effects of exposure to violence and victimization across life domains on adolescent substance use. *Child Abuse Negl.* 37 899–909. 10.1016/j.chiabu.2013.04.010 23743232 PMC4137799

[B100] Yodprang B. Kuning M. McNeil N. (2009). Bullying among lower secondary school students in Pattani Province, Southern Thailand. *Asian Soc. Sci.* 5 23–30.

[B101] Yoon S. Howell K. Dillard R. McCarthy K. S. Napier T. R. Pei F. (2019). Resilience Following Child Maltreatment: Definitional Considerations and Developmental Variations. *Trauma Viol. Abuse* 22 541–559. 10.1177/1524838019869094 31405362

[B102] Yule K. Houston J. Grych J. (2019). Resilience in Children Exposed to Violence: A Meta-analysis of Protective Factors Across Ecological Contexts. *Clin. Child Fam. Psychol. Rev.* 22 406–431. 10.1007/s10567-019-00293-1 30887404

